# Supplementary figures and images for: Single cell transcriptomics reveals dysregulated cellular and molecular networks in a fragile X syndrome model
Source: PLoS Genet. 2022 Jun 8;18(6):e1010221. doi: 10.1371/journal.pgen.1010221 (PMC9212148; doi:10.1371/journal.pgen.1010221)

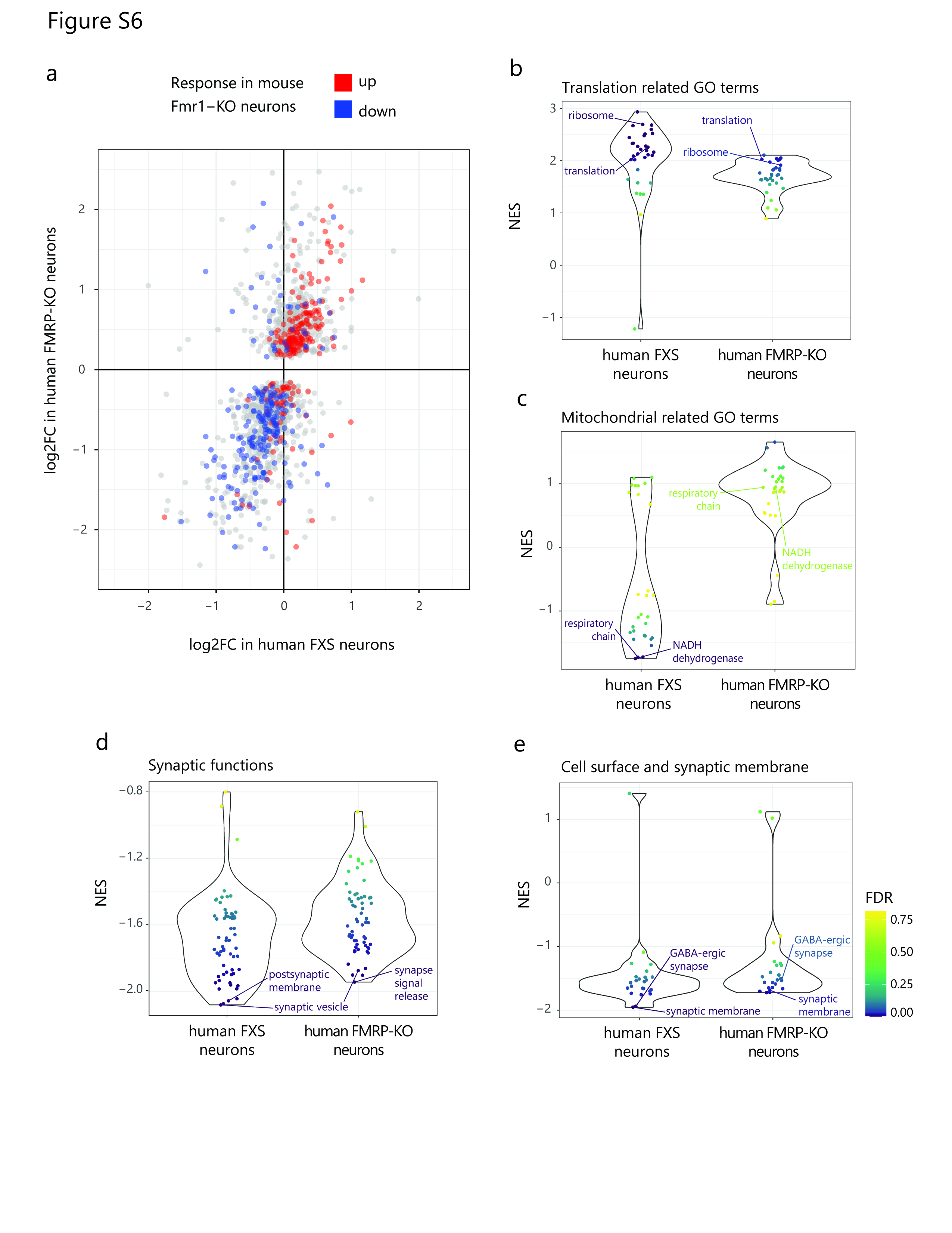

Supplement: S6 Fig — a) log2 fold change relative to healthy human cultured neurons for hiPSC derived FXS neurons and hESC derived FMRP-KO neurons reported in Utami et al. 2020. Genes depicted are homologs to the ones expressed in mouse neurons that show a significant change in one of the cultured human neurons (FDR < 0.01; n = 1953). Red colored genes show a significant upregulation in mouse Fmr1-KO (FDR < 0.01, log2FC > 0), while blue colored genes show a significant downregulation in mouse Fmr1-KO (FDR < 0.01, log2FC < 0). b-e) Comparison of normalized enrichment scores (NES) across human cultured neurons for GO terms involved in different processes which were significantly altered in mouse Fmr1-KO neurons. b) Translational (e.g. Ribosome, GO:0005840; Translation, GO:0006412). c) Mitochondrial (e.g. Respiratory chain complex I, GO:0045271; NADH dehydrogenase complex, GO:0030964). d) Synaptic functions (e.g. postsynaptic membrane, GO:0045211; Synaptic vesicle exocytosis, GO:0016079; Signal release from synapse, GO:0099643). e) GO terms which display opposite signals in mouse neurons and astrocytes (e.g. GABA-ergic synapse, GO:0098982; Synaptic membrane, GO:0097060). (TIF) [file pgen.1010221.s006.tif]
