# Supplementary material for: Single cell transcriptomics reveals dysregulated cellular and molecular networks in a fragile X syndrome model
Source: PLoS Genet. 2022 Jun 8;18(6):e1010221. doi: 10.1371/journal.pgen.1010221 (PMC9212148; doi:10.1371/journal.pgen.1010221)
Supplement: S4 Table — Significant overlaps are shaded in green. (PDF) [file pgen.1010221.s018.pdf]

FMRP target list	DE direction in Donnard et al.	Target list overlap	DE in Donnard et al.	Targets expressed in Donnard et al.	Background genes tested	Hypergeometric p-value
[3] Maurin et al. 2018	down	293	827	1582	7599	1.94E-40
[1] Darnell et al. 2011	down	158	827	698	7599	2.97E-25
[4] Brown et al. 2001	down	61	827	299	7599	1.67E-07
[6] Sawicka et al. 2019	down	50	827	255	7599	8.52E-06
[5] Miyashiro et al. 2003	down	9	827	38	7599	1.78E-02
[5] Miyashiro et al. 2003	up	7	876	38	7599	1.38E-01
[2] Li et al. 2020 vNeuron	down	63	827	546	7599	1.50E-01
[2] Li et al. 2020 dNeuron	down	55	827	490	7599	2.46E-01
[2] Li et al. 2020 vNPC	up	91	876	923	7599	6.89E-01
[2] Li et al. 2020 dNPC	down	80	827	895	7599	8.18E-01
[2] Li et al. 2020 vNPC	down	81	827	923	7599	8.58E-01
[2] Li et al. 2020 dNPC	up	79	876	895	7599	9.48E-01
[2] Li et al. 2020 vNeuron	up	39	876	546	7599	9.99E-01
[2] Li et al. 2020 dNeuron	up	31	876	490	7599	1.00E+00
[6] Sawicka et al. 2019	up	11	876	255	7599	1.00E+00
[4] Brown et al. 2001	up	13	876	299	7599	1.00E+00
[1] Darnell et al. 2011	up	38	876	698	7599	1.00E+00
[3] Maurin et al. 2018	up	89	876	1582	7599	1.00E+00

Reference

[1] Darnell JC, Van Driesche SJ, Zhang C, Hung KYS, Mele A, Fraser CE, et al. FMRP stalls ribosomal translocation on mRNAs linked to synaptic function and autism. Cell. 2011;146(2):247–61.

[2] Li M, Shin J, Risgaard RD, Parries MJ, Wang J, Chasman D, et al. Identification of FMR1-regulated molecular networks in human neurodevelopment. Genome Res. 2020 Mar;30(3):361–74.

[3] Maurin T, Lebrigand K, Castagnola S, Paquet A, Jarjat M, Popa A, et al. HITS-CLIP in various brain areas reveals new targets and new modalities of RNA binding by fragile X mental retardation protein. Nucleic Acids Res. 2018 Jul 6;46(12):6344–55.

[4] Brown V, Jin P, Ceman S, Darnell JC, O'Donnell WT, Tenenbaum SA, et al. Microarray identification of FMRP-associated brain mRNAs and altered mRNA translational profiles in fragile X syndrome. Cell. 2001 Nov 16;107(4):477–87.

[5] Miyashiro KY, Beckel-Mitchener A, Purk TP, Becker KG, Barret T, Liu L, et al. RNA cargoes associating with FMRP reveal deficits in cellular functioning in Fmr1 null mice. Neuron. 2003 Feb 6;37(3):417–31.

[6] Sawicka K, Hale CR, Park CY, Fak JJ, Gresack JE, Van Driesche SJ, et al. FMRP has a cell-type-specific role in CA1 pyramidal neurons to regulate autism-related transcripts and circadian memory. Elife [Internet]. 2019 Dec 20;8. Available from: http://dx.doi.org/10.7554/eLife.46919
