# Supplementary material for: Single cell transcriptomics reveals dysregulated cellular and molecular networks in a fragile X syndrome model
Source: PLoS Genet. 2022 Jun 8;18(6):e1010221. doi: 10.1371/journal.pgen.1010221 (PMC9212148; doi:10.1371/journal.pgen.1010221)
Supplement: Attachment — Submitted filename: PLOSGenetics_response_to_reviewers.pdf [file pgen.1010221.s019.pdf]

We thank the reviewers for acknowledging the value of our manuscript and single cell data as a resource for the FXS research community, and for their constructive comments. We believe that we have satisfactorily addressed all specific issues raised in this review. We address each reviewer's specific comments below:

Reviewer #1:

"Single Cell Transcriptomics Reveals Dysregulated Cellular and Molecular Networks in a Fragile X Syndrome Model" is an interesting manuscript. However, it seems that the authors do not have a clear overview of the Fragile X literature. This – in my opinion – negatively impacts not only the introduction but the analysis of the results and the discussion. My main suggestion is to reconsider all aspects of this manuscript in the light of a larger literature analysis.

For instance:

FMRP is not only a repressor but also an activator of translation: Bechara et al., PLoS Biology 2009; Ethan J. Greenblatt, Allan C. Spradling, Science 2018; Maurin & Bardoni , Front Mol Bioscience 2018.

One pathway that is critically modified in Fragile X is the cAMP-cGMP, as shown by multiple studies: (see a recent selection) Gurney et al., Sci Rep, 2017; Maurin et al., NAR 2018; Maurin et al., Cerebral Cortex 2019; Gurney et al., J Med Chem. 2019; Delhaye & Bardoni, Mol Psychiatry, 2021; Berry-Kravis EM et al., Nat Med, 2021

If the authors have results supporting these findings they have to underline them, if they do not have them, it is a critical point to be discussed.

We thank the reviewer for bringing these relevant studies to our attention. We have included the references on FMRP being a translation activation, and those on FMRP regulating PDE2A and the cAMP-cGMP pathway to the introduction. We share the excitement of the reviewer with regard to the potential of targeting PDE as a therapeutic avenue as shown by the reference suggested by the reviewer in which inhibition of Pde2a or Pde4d enzymes show social and cognitive improvement in both the mouse model of FXS and in FXS patients. Similarly we agree that cAMP dysregulation has been characterized decades ago (Berry-Kravis and Huttenlocher 1992).

Our data shows a very low expression level and no significant change in Pde2a mRNA in the *Fmr1*-KO cortical neurons (FDR = 0.012; Reviewer Figure 1A), nor in the cAMP-mediated signaling pathway (FDR = 0.039; GO:0019933; Reviewer Figure 1B) mRNAs. This can likely be a reflection of the developmental stage, and the fact that FMRP regulates the phosphodiesterase encoding mRNA as well as cAMP metabolism at the translation and protein signalling level, but not at the RNA level.

Reviewer Figure 1

A B

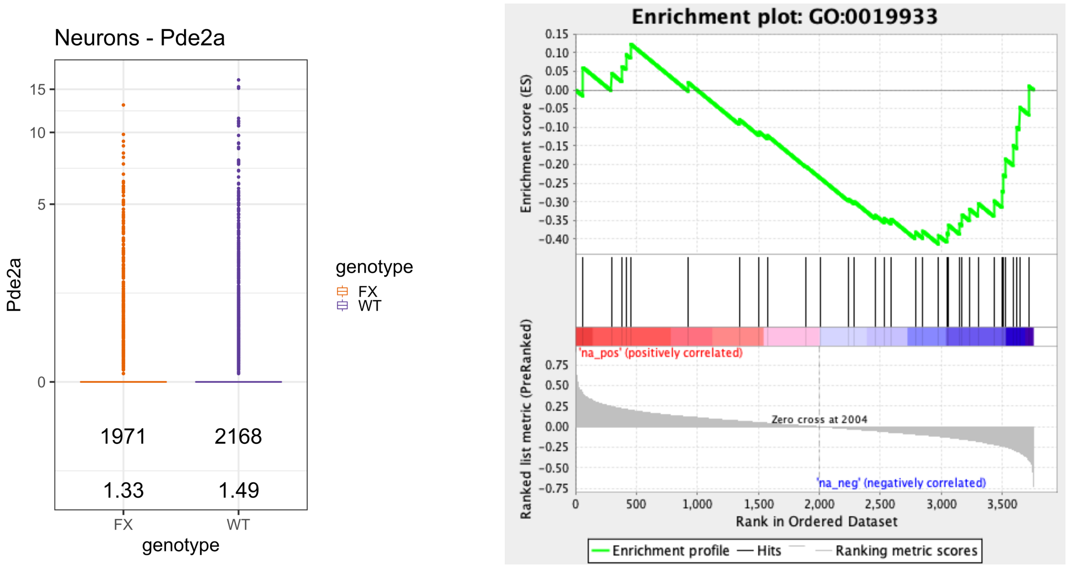

Reviewer Figure 1

A) Expression in all mouse neuronal cells for Pde2a, numbers at the bottom indicate number of cells in each group, and the median UMI expression. **B)** GSEA enrichment plot showing expressed genes in mouse neurons ranked by their fold change between *Fmr1*-KO and WT. Black bars indicate genes that are present in the cAMP-mediated signalling (GO:0019933).

The implication of FMRP in excitatory/inhibitory balance has been already considered in another study using single-cell RNAseq: Castagnola et al., Genome Res., 2020. These data should be mentioned by the authors who, in addition, should compare they results with those of Castagnola et al and discuss them.

We thank the reviewer for bringing this publication to our attention. Castagnola *et al.* (2020) showed a global altered AMPA response in Fmr1-KO excitatory vs inhibitory neurons. AMPA receptor subunits need to be inserted to the postsynaptic membrane by vesicle trafficking factors such as Vamp2 to regulate the excitatory synapses (Hussain and Davanger 2015). We show that in FK neurons, the Vamp2 mRNA was dysregulated (Figure S6a), which could lead to impaired trafficking of AMPA receptors, and in turn contribute to the excitatory-inhibitory imbalance in the FXS brain.

We have included this reference in the results section where Vamp2 is discussed, as well as in the Discussion section.

Several previous analyses have defined by CLIP the target mRNAs of FMRP (please see for an exhaustive list in Richter JD & Zhao X Nat Rev Neurosci. 2021). It would be important to compare mRNAs whose expression is found altered here by Donnard et al., with the various lists found by different authors in order to understand :

i) The possible mechanism of action of FMRP;

ii) The alterations that are directly caused by the absence of FMRP and those that are due to a “cascade” effect.

The mRNA binding targets of FMRP have indeed been assayed in multiple tissues and organisms (Richter and Zhao 2021), and the reason we decided to focus on the mRNAs detected by Darnell et al. were primarily due to 1) the more stringent technique used (HITS-CLIP) with a smaller list of potential binding targets (842) compared to many other more recently published, 2) the fact that it was performed using a similar mouse model as our own study with a closer age included in the study (P14/P15), and 3) the large overlap between this list and other related mouse-based target lists. However, we agree with the reviewer that there is added value in reporting the overlap between the genes we see as differentially expressed and other putative FMRP binding target lists. To this end, we report here the overlap between the genes we detect as DE and several of the lists included in the cited review (Brown et al. 2001; Darnell et al. 2011; Maurin et al. 2018; Li et al. 2020). We focused on the studies that were specifically trying to identify mRNAs bound by FMRP, and that assay human neurons and mouse cortex as opposed to other brain regions or unrelated cell types. Below are the enrichments for the overlap with each of these lists.

FMRP target list	DE direction in Donnard et al.	Target list overlap	DE in Donnard et al.	Targets in list expressed in Donnard et al.	Background genes tested for DE	Hypergeometric p-value
Brown 2001	down	61	827	299	7599	1.67E-07
Darnell 2011	down	158	827	698	7599	2.97E-25
Maurin 2018	down	293	827	1582	7599	1.94E-40
Li 2020 dNeuron	down	55	827	490	7599	2.46E-01
Li 2020 dNPC	down	80	827	895	7599	8.18E-01
Li 2020 vNeuron	down	63	827	546	7599	1.50E-01
Li 2020 vNPC	down	81	827	923	7599	8.58E-01
Brown 2001	up	13	876	299	7599	1.00E+00
Darnell 2011	up	38	876	698	7599	1.00E+00
Maurin 2018	up	89	876	1582	7599	1.00E+00
Li 2020 dNeuron	up	31	876	490	7599	1.00E+00
Li 2020 dNPC	up	79	876	895	7599	9.48E-01
Li 2020 vNeuron	up	39	876	546	7599	9.99E-01
Li 2020 vNPC	up	91	876	923	7599	6.89E-01

As shown in the table above, only three lists, those that assayed the mouse cortex, have a significant overlap with the downregulated genes we detected. Additionally, it was previously known that a significant fraction of the targets are common between these three lists (Brown, Maurin and Darnell), and therefore we think this result is expected. In the Reviewer Figure 2A and 2B below, we display genes that we detect as upregulated (A) or downregulated (B) in at least one cell type, and their presence in any of these lists or none of them (NA). The overlap results don't point to a particular mechanism, however, it is encouraging to see that many of these critical neuron development genes overlap, and the enrichment for multiple of these target lists provides further evidence that the downregulation we observe in Fmr1-KO neurons is a direct effect from the absence of FMRP, as we suggested in the text considering the overlap with the Darnell list. We thank the reviewer for this suggestion and have included this enrichment table as a supplemental material.

Reviewer Figure 2

A - Upregulated genes in our mouse scRNA-Seq data present in each tested list of FMRP bound mRNAs. Genes that overlap none of the lists are in the NA category.

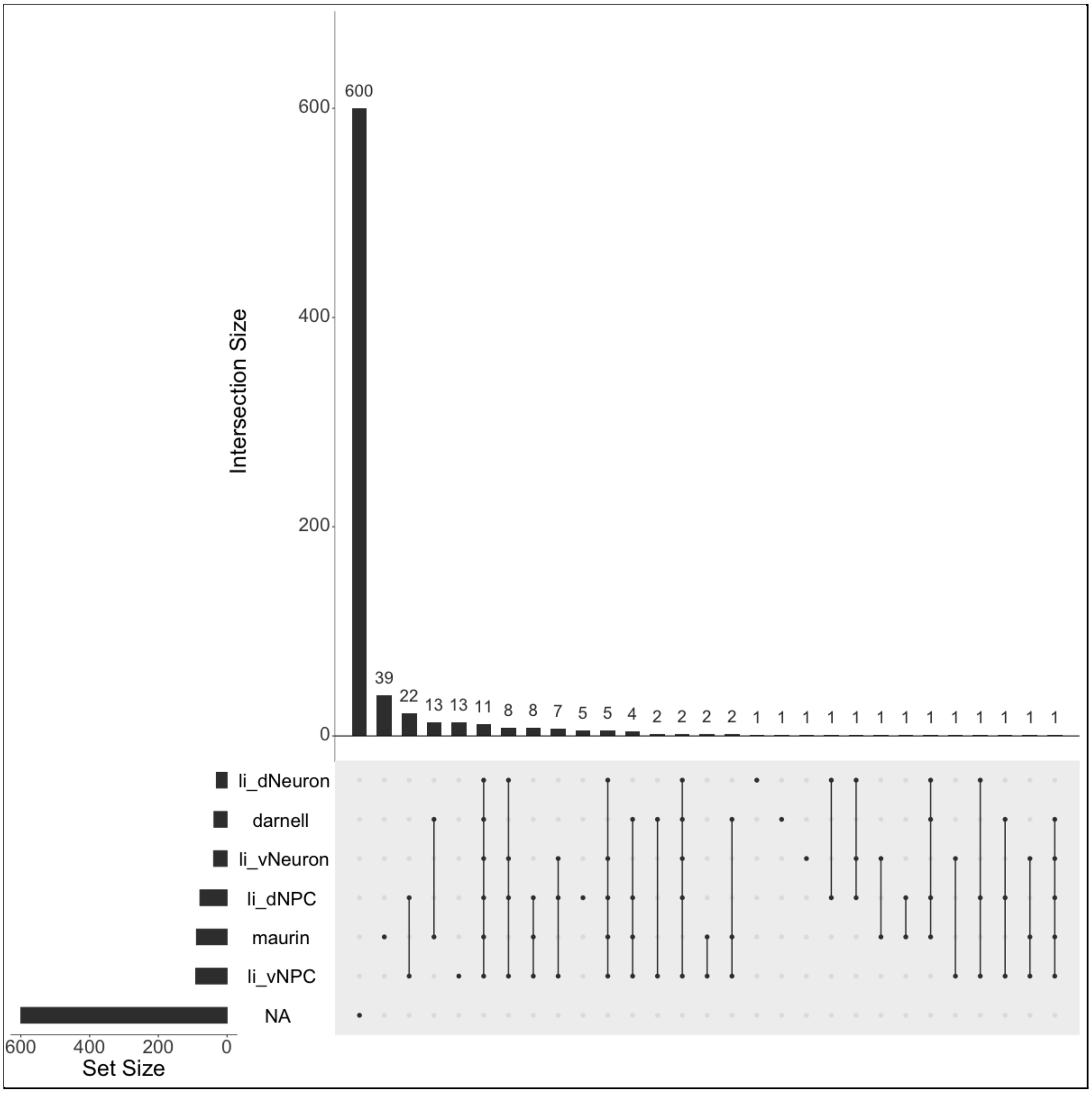

B - Downregulated genes in our mouse scRNA-Seq data present in each tested list of FMRP bound mRNAs. Genes that overlap none of the lists are in the NA category.

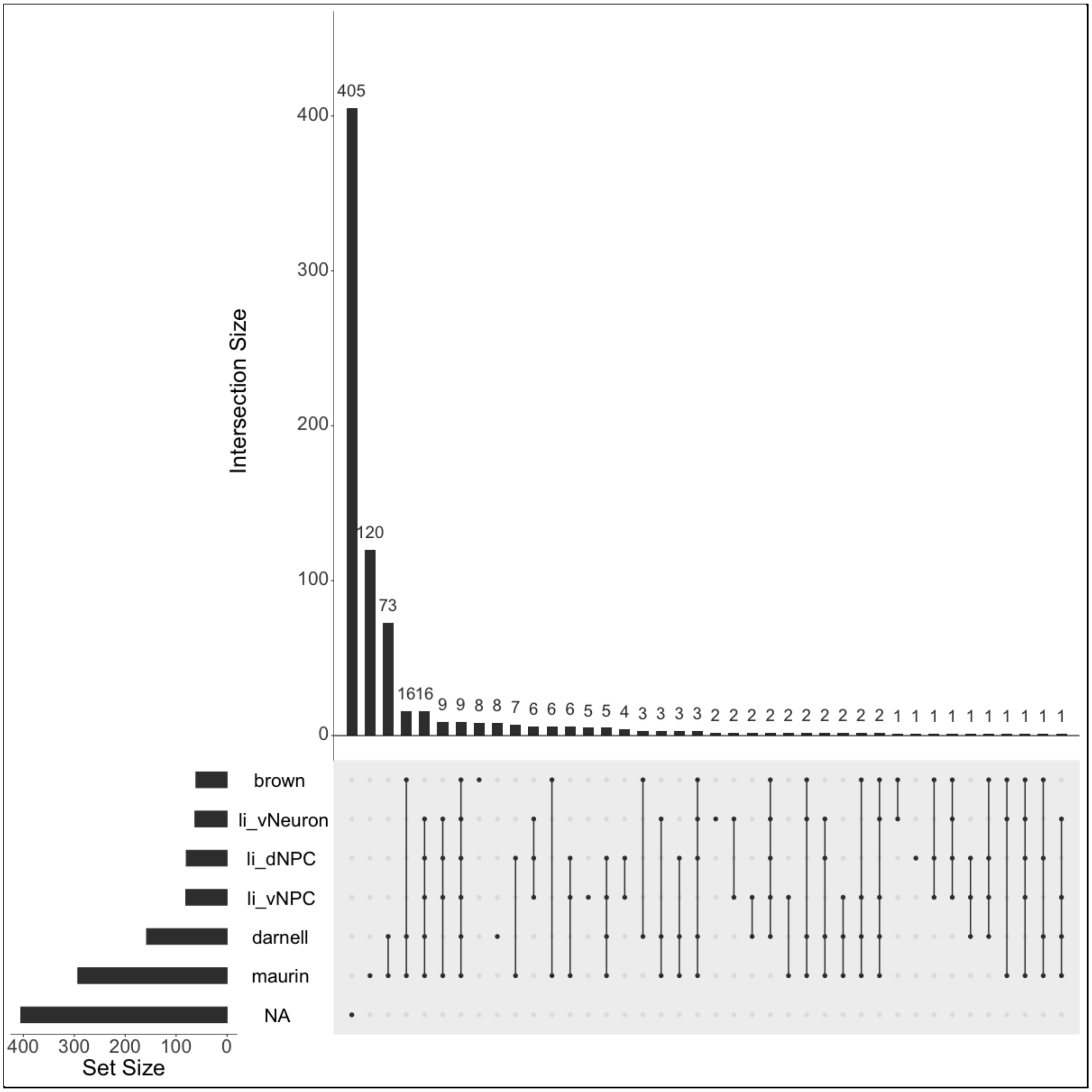

-The mechanism of action of FMRP is quite complex, that seems here oversimplified

We apologize that our wording gave the reviewer a wrong impression that we are oversimplifying the mechanism of the action of FMRP. We discussed a complex array of possible mechanisms of action via which FMRP could impact the various stages in the life cycle of its target mRNAs in the Introduction section. The data presented in our manuscript focuses on the description of the complicated transcriptomic consequences in the various cell types in the FK mouse brain, without trying to speculate about the mechanisms that result in these changes. We reworded the Discussion section to emphasize that while our data is consistent with FMRP loss resulting in decreased RNA stability, which would explain the general trend in downregulation of FMRP target mRNAs in neurons, we now explicitly mention other possible mechanisms.

In the discussion the authors should underline the novelty of their study not only as a technological approach but at the level of study of the pathways involved in the pathophysiology of Fragile X. For instance, Figure 5 illustrates findings already known 5-6 years ago.

We thank the reviewer for this suggestion. We reworked our discussion to better highlight novel pathways involved in the pathophysiology of Fragile X. Specifically, we highlighted the three major findings and discussed them in detail: i) Higher impact of FMRP loss in neurons; ii) How loss of FMRP results in cell type specific differences in mTOR activity; iii) Changes in abundance of cell-cell signalling genes that could increase environmental excitability. We modified the final paragraphs of the discussion to better highlight the novelty of our data in terms of the biological significance.

However, we respectfully disagree with the reviewer's comment about Figure 5 illustrating findings already known 5-6 years ago. Actually, we are uncertain as to what exactly the reviewer considers as known 5-6 years ago. Figure 5 shows GO categories (Figure 5a) or individual genes present in these categories (Figure 5b-c) that we identified as having changed in *Fmr1*-KO cortex in a cell type specific manner that could impact cell-cell communications. The functions of these pathways are described in previous literature and are cited in the text. However, in Figure 5, we highlight expression changes of these functionally related genes that we identified. These changes are described for the first time in our manuscript, and we specifically cite the previous FXS related research that reported many pathological phenotypes that could be tied to the changes we see.

Minor: please correct polysome with polyribosomes

We have changed "polysome" to "polyribosome" in our manuscript as recommended.

Reviewer #2:

In this manuscript, the authors used single cell RNA sequencing to profile an FMRP deficient mouse model for Fragile X syndrome. Their findings suggest that FMRP loss affect cell-cell communication, thus resulting in a cortical environment of greater excitability. The study provides an useful resource for investigating molecular basis of Fragile X syndrome.

While the data are interesting, they are solely transcriptomics (mRNA) based. For many of the dysregulated genes, such like Ephb3 and Epha4 (upregulated in astrocytes), downregulation of Slc1a4 in neurons, upregulation of Gabbr1 and Gabbr2 in Fmr1-KO astrocytes, Slc6a1 (GAT-1), and Slc6a9 (GlyT-1) upregulated in astrocytes, downregulation of a GABAA receptor gamma subunit (Gabrg2) and reduced expression of Slc12c5 (KCC2) in Fmr1-KO neurons, the authors will need to use additional approach (immunocytochemistry, Western blot or others) to validate or assess the gene expression changes at protein levels. This is to further confirm that the dysregulated gene expression is able to reach the protein level to function biologically.

We thank the reviewer for the interest in our data and further interest in seeing some of the mRNA level changes in individual genes validated at protein level. We agree in principle with the value in validating changes at protein level for our insight into the FXS. Although we had foreseen the challenge in achieving this due to the cell type specificity of these effects as well as the small magnitude nature of most of these changes we found, we attempted validating the protein level changes of Mt1/Mt2, the strongest changed genes in all the DE genes, which are downregulated in astrocytes specifically (log2FC = -0.7, and have been validated

using RT-qPCR in independent samples; Reviewer Figure 3). We tested two antibodies against MT1/MT2 in western blots with cortical tissue lysates or with primary cultured astrocyte lysates, as well as immunostaining in cortical slices. Unfortunately the antibodies we tested were too unspecific to lead to any conclusions, and no others were available with higher quality.

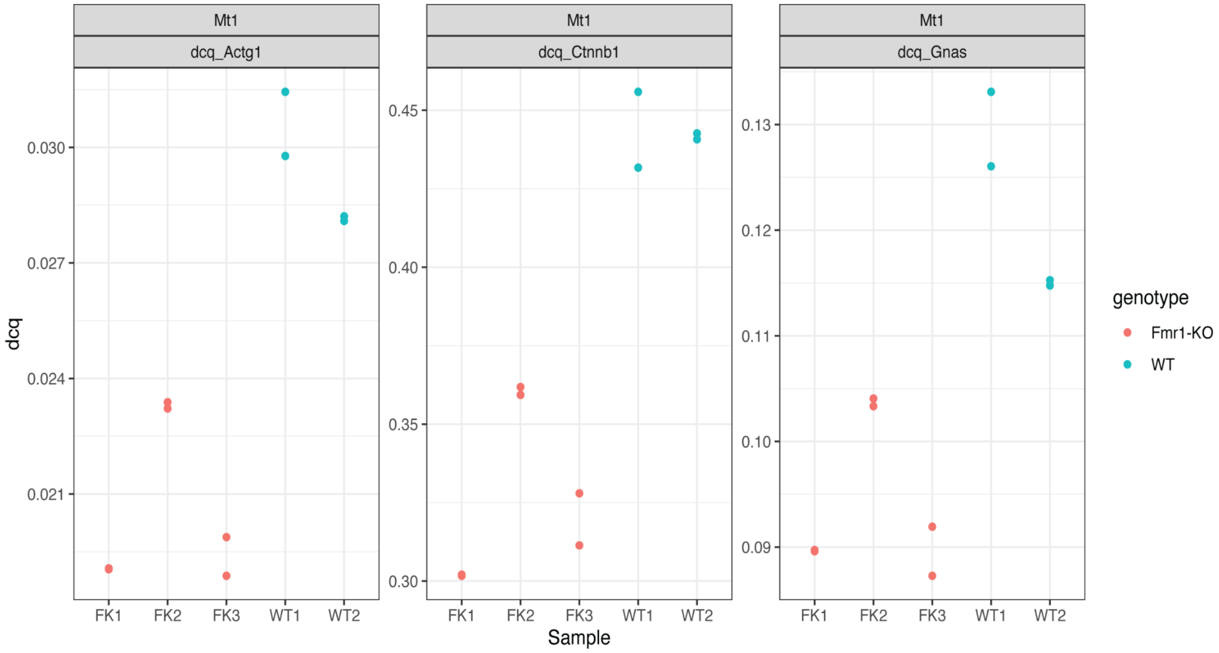

Reviewer Figure 3 - Mouse whole cortex qPCR results for Mt1 expression. Delta Cq values are shown normalized to three different control genes (Actg1, Ctnnb1, Gnas). Samples FK1, FK2 and FK3 indicate three different *Fmr1*-KO mice, Samples WT1 and WT2 indicate two different WT mice, individual dots indicate technical replicates of the qPCR reaction.

On the other hand, the novel finding in our manuscript is that in the *Fmr1*-KO cortex multiple pathways are changed, and corresponding genes within are changed collectively in the same direction in a cell type-specific manner. We hypothesize that it is this collective change that drives the pathophysiology rather than specific genes being affected. We hope that the reviewer agrees with us in that confirming that pathways are affected in a cell type specific way at protein level is outside the scope of this study. We have included this point in the Discussion.

Nevertheless, as the reviewer points out, our transcriptomic data showing the cell-specific pathway level changes, provides a valuable resource that can encourage a new perspective in understanding the FXS and open up new research avenues for this disease.

Reviewer #3:

Donnard et al performed single cell RNA sequencing (scRNA-seq) with postnatal day 5 Fmr1 KO cerebral cortex to understand the mechanisms by which Fmr1 regulates FXS. The analyses generated some useful information, but this reviewer finds that the manuscript needs to provide rigorous statistical improvement and more experimental results.

1. To get more comprehensive results, the sc-RNA seq should be also done with brains at more mature stage at least 4-8 weeks old. Postnatal day 5 is estimated as gestational week 21 (Clancy, 2007). The usual diagnosis time and the average age of first concern is 12 months based on the CDC report. The authors could show if the DE patterns are conserved

or changed. If changed, what would be the functional relationship between the DE and the phenotypes?

We appreciate the reviewer's point. While it is true that the disease in humans is clinically diagnosed at a later age compared to the one we have examined, our study focused on identifying molecular phenotypes that are present even if sub-clinically at earlier developmental stages. Our goal was to identify molecular changes that precede disease onset and that therefore may eventually help to provide novel therapeutic clues. To this end, we focused on postnatal day 5, which is a known “critical window” in mouse cortical development, as evidenced by many other studies (Farhy-Tselnicker and Allen 2018; Harlow et al. 2010; Nomura et al. 2017), and which has for that same reason been the focus of FXS related studies on which we based ours (Darnell et al. 2011). While a study that looks at later stages of brain development would also be fascinating, our goal was to focus on the effects of FMRP loss on the developing brain with the goal to open new avenues of investigation and generate a resource that new studies can build upon. We have better explained our motivation for looking at the developing brain in Discussion.

2. ìWe examined differential expression between Fmr1-KO and WT mice for each cell type identified. In total, we identified 1470 differentially expressed (DE) genes (FDR < 0.01 and fold change >=1.15) in one or more cell types. The majority of DE genes showed small fold changes (mean fold change = 1.3x).î

This reviewer is most concerned about getting conclusion with the DE with fold change >=1.15. With such a small fold change, many of them just could be from biological sample or experimental batch variations.

The fascinating observation of ours and other studies is that loss of FMRP does not cause a large effect on the mRNA levels of most genes, however, the effect is consistent across most genes within affected pathways. We did not try to make claims about any particular gene and in fact, we agree with the reviewer in that conclusions about any particular gene may be challenging at this level, and only applied this low threshold to define the numbers in Figure 2a. Our analysis instead focused either on specific genes with larger fold changes or on pathways or gene sets with similar function where the overall trend is highly significant. The overall small effect size of FMRP loss was one of the motivations of carrying out a single cell analysis of FXS loss of function. In this version we have made an effort to better make this point in the introduction: that the small effect size of the transcriptional changes detected is expected, given that more robust changes would have been observed also by previous studies which relied on bulk analysis of gene expression.

We want to stress that our approach to look at functionally related genes using gene set enrichment analysis (GSEA) does not rely on a cutoff for selecting differentially expressed genes, but instead examines the fold changed ranked gene list to identify any coordinated expression change involving genes that belong to the same pathways or categories. As a result, the genes in the pathways identified show a consistent (and significant compared to other lists of genes) up or down regulation, which is a strong indicator of a biologically relevant change resulting from the loss of FMRP.

3. Given the nature of the data with such a small DE FC, the authors should show separate tSNE plots for Wt versus KO in all tSNE related figures. It wonders whether there is no distinct cell cluster(s) in fmr1 KO compared to control. Even with overall similar presentation

in tSNE plot, Wt and KO could show some differential proportion change better if they are presented separately.

The reviewer raises a valuable question, we did in fact provide a figure of the tSNE representation of the cells colored by their genotype in Figure S1b. As we discussed in the text, there were no observable differences or clusters that were formed by cells of only one genotype. This is not encouraging, considering the analysis relies on a set of genes that exhibit the highest variability across cells, and this is largely dominated by the cell type specific signal. Likewise, we examined these genotype plots for every subclustering analysis performed, using each cell type independently, and found no consistent differences that signalled a genotype difference in proportions. Again, this is not surprising given that the changes we observed in expression are moderate. We provided both the code to generate these plots (https://github.com/elisadonnard/FXSinDrop) as well as the single cell matrix used as input (GSE147191).

4. Throughout the entire manuscript, the authors should provide the adjusted p values or FDR values of the DE genes. The authors provided p-vlaue for enrichment analysis, but did not mention statistical information for the analysis with differential expression genes. Without the proper significance, the claim of the authors could not be reasonable. The authors adopted FDR<0.25 0r 0.3 in many places.

Unless specified, we used an FDR of 0.01 or less in our comparisons. However, as the reviewer points out, we did in some cases want to point out specific examples or trends that were significant in one cell type but not others, yet the trends were similar. Specifically:

- In figures describing GSEA results we report all categories that reach our FDR level **in at least** one cell type, but also report their enrichment and FDR in all other cell types (e.g. Figure 2b legend, where we specifically say the FDR<0.3 colors refer to trends in other cell types). This is the same reason why categories reported in Figures 2c-d, 4, 5a, S5b-4 and S9c-e) may not reach the 0.01 cut-off for some cell types. Every category has reached this significance in at least one cell type. We hope that the reviewer agrees with us that it is informative to report how every category that is disrupted in one cell type is affected on the other cell types.
- When we show the GSEA results for synapse related pathways (which are significantly downregulated in all neurons) in excitatory and inhibitory neuron subtypes. This panel is intended to show a similar trend between the two neuronal subtypes, without claiming the significance of the change. We have made this clearer in the text as well.
- When we discuss potential mechanisms for disruption of mTOR activity (Discussion) we mention that Grin2b is upregulated in excitatory neurons (FDR = 0.019). We explicitly point out that this gene is upregulated (revised Figure S12b) but it just barely misses our significance cut-off.

5. Most of the violin plots in Figure S2d,e and S4c, do not look differential and significant to me. The results of bulk RNA-seq data seem to be driven by one very high outlier. The current violin plot is distracting since the outlier drags the boxes very high. I would suggest to use a boxplot to re-draw the results.

We thank the reviewer for raising the concern that for the individual genes shown in Figure S4, violin and point plots are difficult to read, and therefore we have changed to boxplots as recommended. These genes are indeed significantly different between the WT and FK,

although not all of them show a large degree of change - the dominant majority of the DE genes as we have discussed show moderate fold changes. Nevertheless, we chose to display the comparison of these individual genes as a few examples from the more relevant Gene Ontology categories, so that the readers could associate these pathways to key genes of interest.

With respect to the human bulk RNA-Seq data (now Figure S6b), which are sourced from (Utami et al. 2020) (the citation is included in the legend for better clarification), we agree that one WT sample seems to be an outlier and shows much higher expression than other WT samples for the genes shown. However, after removing this outlier, there is still a considerable reduction in expression of these genes in the FXS neurons (average log2 fold change -0.7), and more pronounced in the FMRP-KO derived neurons.

6. In Figure 4a, if we look at the terms with FDR<0.05, excitatory and interneuron do not show much difference.

We thank the reviewer for making an insightful observation. It is indeed our intention to show that for the synaptic related GO terms (Figure 4a), excitatory and inhibitory neurons behave very similarly. This is intended to contrast with the divergent behavior of the excitatory and inhibitory neurons for translation and mitochondria related GO terms (Figure 4b-c). We intend to show GO terms that behave similarly and differently to present this overview that excitatory and inhibitory neurons share some common responses but also have some divergent responses. However we have moved Figure 4a to 4c, and modified the text of this section to further clarify the intention.

7. In many places, the authors did not present Wt versus KO data side by side, which would not tell better the differential effect by loss of FMRP. For example, the authors should show the developmental change in the expression of specific subset of genes in both Wt and KO in Figure S6d, e and f or DE along with significant p-value. The authors should already have these data and could utilize the data fully to claim better. I believe people in FXS research would be more interested in the changes happening in KO.

We thank the reviewer for the interest in the expression profile of FMRP bound mRNAs over the developmental stages in the *Fmr1*-KO brain as we compiled and showed for WT in the current FigS11a-b. Please note that the expression data of these genes in the WT mouse brains were compiled from multiple published data sets (as cited in the main text), and the corresponding *Fmr1*-KO data does not exist. Our intention with this analysis was to point out potential avenues of discovery, but we do not make any claim for differences between *Fmr1*-KO and WT. We agree that this would be interesting for researchers in the FXS field and tried to highlight it by showing evidence for striking temporal patterns in the expression of these genes.

8. ìThe cell type specific alteration of the transcriptome is a sensitive reflection of the cellular status, and can serve as a first step towards an overview of the molecular impact of FXS.î If this is the purpose of this manuscript, cell-type specific KD of Fmr1 including neuron, endothelial cell and astrocytes should be shown. It is likely that the observed DE patterns of this manuscript is combined effects of cell intrinsic and extrinsic functions of Fmr1 and/or combined effects of multiple cells lacking Fmr1. Some experimental verification that can support the authorsí conclusions should be presented. Otherwise, enthusiasm on this manuscript would be minimal.

We thank the reviewer for affirming the significance of our data as a sensitive reflection of the cellular status for the mouse model of FXS. The pathophysiology of the FXS is complicated and involves the interaction of numerous cell types and pathways. Our data presents the first of such with an overview, and we believe it will prove a valuable resource for the FXS field, the field of neurodevelopmental diseases, as well as the field of neurodevelopmental biology as a whole. And, as with the FXS disease, FMRP is lost in all cell types, the cellular status reflected by the transcriptome changes could be due a combination of both intrinsic and extrinsic disturbances. Indeed it would be an important next step to compare the transcriptomic changes in cell type specific KO models, in order to parse out the cellular intrinsic and extrinsic mechanisms. We agree that validation of our conclusions would increase the impact of our study, however, given that this is the first study that dissects cell type specific effects on mRNA levels in-vivo for the *Fmr1*-KO mouse, we believe that validation work will be critical and we are sure that this study will motivate them. Nevertheless, we thank the review for this insight and have added this point to the Discussion.

9. The arrangement of Supplemental figures is not easy to follow. The orders of Supplemental figures should go in parallel with the main text.

We agree with the reviewer and have reordered the Supplemental figures.

10. Some of the figures and Supplemental figures were not cited in the main text, for example, Figure S4a.

We thank the reviewer for pointing out this missing citation and have corrected it in the current text.

11. There is no Figure 2f in Figure 2, which is cited in the main text

We thank the reviewer for pointing out this typo and have corrected it in the current text.

12. There is no reference (Utami et al, 2020)cited in the Suppl figure legend. If the data was from Utami, 2019, the cells must be hESC, not iPSC derived neuron.

We thank the reviewer for pointing out this citation was not up to date and have corrected it in the current text. As for the correction from hiPSCs to hESCs, we have clarified the mentions in our text, as both types of cell are present in the Utami et al. 2020 study, with the FMR1-KO neurons being generated from hESCs and the human FXS neurons derived from iPSCs.

13. The comparison between mouse brain and human should be with human post mortem or at least forebrain organoids at comparable developmental stage.

We agree with the reviewer that transcriptomic data from human FXS *in vivo* neurons, or single cell data from post mortem cortex, would be ideal for comparison. However, to the best of our knowledge, these data do not exist. However, in the past month (after our manuscript had been reviewed), Kang *et al.* published the first ever human FXS organoid model (Kang et al. 2021). Kang *et al.* present evidence for enhanced neuronal excitability through immunocytochemistry, and in agreement with our results they report downregulated neurodevelopmental pathway genes, as well as upregulated protein translation and mitochondrial pathway genes by single cell RNA-seq, albeit without defining the cell types in which such dysregulation happens. The conclusions by Kang *et al.* strongly corroborate our findings. In return, our data complements that of Kang's with unparalleled cell type resolution

(for example, as shown in Fig 4, the excitatory and inhibitory neurons exhibit a change in opposite direction for translation and mitochondrial pathways, which Kang *et al.* mention only as upregulated). We believe our data combined with studies such as these will provide invaluable resources for the FXS research community. We have added the above to Discussion.

REFERENCES

Berry-Kravis, E., and P. R. Huttenlocher. 1992. "Cyclic AMP Metabolism in Fragile X Syndrome." *Annals of Neurology* 31 (1): 22–26.

Brown, V., P. Jin, S. Ceman, J. C. Darnell, W. T. O'Donnell, S. A. Tenenbaum, X. Jin, et al. 2001. "Microarray Identification of FMRP-Associated Brain mRNAs and Altered mRNA Translational Profiles in Fragile X Syndrome." *Cell* 107 (4): 477–87.

Darnell, Jennifer C., Sarah J. Van Driesche, Chaolin Zhang, Ka Ying Sharon Hung, Aldo Mele, Claire E. Fraser, Elizabeth F. Stone, et al. 2011. "FMRP Stalls Ribosomal Translocation on mRNAs Linked to Synaptic Function and Autism." *Cell* 146 (2): 247–61.

Farhy-Tselnicker, Isabella, and Nicola J. Allen. 2018. "Astrocytes, Neurons, Synapses: A Tripartite View on Cortical Circuit Development." *Neural Development* 13 (1): 7.

Harlow, Emily G., Sally M. Till, Theron A. Russell, Lasani S. Wijetunge, Peter Kind, and Anis Contractor. 2010. "Critical Period Plasticity Is Disrupted in the Barrel Cortex of FMR1 Knockout Mice." *Neuron* 65 (3): 385–98.

Hussain, Suleman, and Svend Davanger. 2015. "Postsynaptic VAMP/Synaptobrevin Facilitates Differential Vesicle Trafficking of GluA1 and GluA2 AMPA Receptor Subunits." *PloS One* 10 (10): e0140868.

Kang, Yunhee, Ying Zhou, Yujing Li, Yanfei Han, Jie Xu, Weibo Niu, Ziyi Li, et al. 2021. "A Human Forebrain Organoid Model of Fragile X Syndrome Exhibits Altered Neurogenesis and Highlights New Treatment Strategies." *Nature Neuroscience* 24 (10): 1377–91.

Li, Meng, Junha Shin, Ryan D. Risgaard, Molly J. Parries, Jianyi Wang, Deborah Chasman, Shuang Liu, Sushmita Roy, Anita Bhattacharyya, and Xinyu Zhao. 2020. "Identification of FMR1-Regulated Molecular Networks in Human Neurodevelopment." *Genome Research* 30 (3): 361–74.

Maurin, Thomas, Kevin Lebrigand, Sara Castagnola, Agnès Paquet, Marielle Jarjat, Alexandra Popa, Mauro Grossi, Florence Rage, and Barbara Bardoni. 2018. "HITS-CLIP in Various Brain Areas Reveals New Targets and New Modalities of RNA Binding by Fragile X Mental Retardation Protein." *Nucleic Acids Research* 46 (12): 6344–55.

Nomura, Toshihiro, Timothy F. Musial, John J. Marshall, Yiwen Zhu, Christine L. Remmers, Jian Xu, Daniel A. Nicholson, and Anis Contractor. 2017. "Delayed Maturation of Fast-Spiking Interneurons Is Rectified by Activation of the TrkB Receptor in the Mouse Model of Fragile X Syndrome." *The Journal of Neuroscience: The Official Journal of the Society for Neuroscience* 37 (47): 11298–310.

Richter, Joel D., and Xinyu Zhao. 2021. "The Molecular Biology of FMRP: New Insights into Fragile X Syndrome." *Nature Reviews. Neuroscience* 22 (4): 209–22.

Utami, Kagistia Hana, Niels H. Skotte, Ana R. Colaço, Nur Amirah Binte Mohammad Yusof, Bernice Sim, Xin Yi Yeo, Han-Gyu Bae, et al. 2020. "Integrative Analysis Identifies Key Molecular Signatures Underlying Neurodevelopmental Deficits in Fragile X Syndrome." *Biological Psychiatry* 88 (6): 500–511.
