# Supplementary material for: Single cell transcriptomics reveals dysregulated cellular and molecular networks in a fragile X syndrome model
Source: PLoS Genet. 2022 Jun 8;18(6):e1010221. doi: 10.1371/journal.pgen.1010221 (PMC9212148; doi:10.1371/journal.pgen.1010221)
Supplement: Attachment — Submitted filename: PLOS_Genetics_round2_responses.pdf [file pgen.1010221.s020.pdf]

We appreciate the reviewers' feedback and response to our comments. Below we address any open comments:

Reviewer #1:

1) In this manuscript the authors profiled by single cell RNA-seq WT and Fmr1-KO mouse brain cortex at postnatal day (PND) 5. They found a very high heterogeneous expression of target mRNAs of FMRP depending on the cell type. Very likely, the authors analyzed by single cell RNA-seq mouse brains at PND 5 for technical reasons (neuronal dissociation is easy at that age). Since it is known that the highest expression of FMRP is between 7 and 14 PND, I would suggest to tone down the discussion about the importance of post-natal-day 5 in FXS (pag. 12).

We appreciate the reviewer's concern. Indeed there are technical considerations in choosing to work with postnatal day 5 for the exact reason the reviewer had mentioned. However, that was not the sole reason we chose to use this time point. For example, we did not choose to work with earlier postnatal days or embryonic brains that are even easier to dissociate, due to the biological relevance of P5, i.e., it being a critical period for mouse cortical development, as well as it being prior to when most behavior phenotypes are detected. Notably, we are not trying to argue that P5 is any more important than other developmental stages, as is discussed in page 12, "*It is possible that different FMRP bound mRNAs are downregulated at different developmental stages in mouse brains that lack FMRP, and that a different set of FMRP bound mRNAs may become of more relevance to the pathology at adult stages (Figure S11a-b).*".

Nevertheless it would be indeed a very valid point that the reviewer raised if FMRP is expressed lower in P5 than P7-P14. We went through the literature trying to find publications that had shown this data, but unfortunately we could not find any. The Kind lab showed lower FMRP level in adult mouse brains than those of P7 and P14, but did not test earlier age (Till et al. 2012). Using immunocytochemistry and semi-quantitative analysis, Gholizadeh et al. estimated about the same levels of FMRP protein at P0 and P10 in neurons, where FMRP is highest expressed (Gholizadeh, Halder, and Hampson 2015). If the reviewer could kindly share the reference showing higher FMRP levels at P7 than earlier stages, we will happily cite it and adjust our text accordingly.

2) Furthermore, they compared their RNA dataset with FMRP RNA targets obtained in 1-month old mouse brain (Darnell et al., 2011). I consider this as an inconsistency since other studies identified FMRP RNA targets (or proteins whose expression is linked to the presence of FMRP, e.g. Tang et al., Proc Natl Acad Sci U S A. 2015 112(34):E4697-706) during the earliest phases of development. For this reason, at the first round of revision I asked to consider other datasets of FMRP targets in addition to the one they used (Darnell et al., Cell 2011). This was only superficially done in the revised manuscript by adding the incomplete table S4. This table is incomplete since, for instance, two important studies such as Sawicka et al, Elife (2019)n8:e46919 and Miiyashiro et al., Neuron (2003) 37(3):417-31 are missing. Please add those datasets to your Table S4.

We appreciate the reviewer's suggestion and have included these two studies in the mentioned comparisons as well.

3) The same in figure S8 a-b, please repeat the analysis shown with datasets other than the one published in 2011 by Darnell.

We have displayed the cdf distributions shown in Figure S8 for the other gene lists included in Table S4, and also included the same for the mouse FMRP-KO neurons. This is now included as Figure S10.

4) Description of Supplementary Tables is just minimal, a lot of abbreviations are not explained in the legend. For instance, in Table S1 what does clip mean? Cross-linking ImmunoPrecipitation? In this case, please indicate results of each clip as in Table S4 and in Figure S8.

We thank the reviewer for pointing out these unclear column descriptions, and have modified the Table S1 and S2 legends to make them more easy to interpret, including the full descriptions from edgeR and GSEA reports, as well as explanations for the columns we created during our analysis. We have also included other target gene lists in Table S1 for genes previously identified as bound by FMRP.

5) The references' list is totally confused and imprecise. Many papers mentioned in the text or in the supplementary material do not appear in the final list. 3 examples:

Li, et al., (2020) Genome Research 30 (3): 361–74.
Castagnola et al. (2020) Genome Research 30(11):1633-1642.
Ritcher & Zhao (2021) Nat Rev Neurosci, 22(4):209-222

We thank the reviewer for pointing this out and have included these citations which refer to the new Table S4, as well as a reference for the table itself in the manuscript.

6) While other articles present in the reference list are not mentioned in the text. 2 examples:

Gurney et al., (2017) Scientific Report, 7 (1) 14653
Berry Kravis et al., (2021) Nature Medicine, 27 (5): 862–70.

These two articles were correctly mentioned and cited in the introduction section of the manuscript, on the second paragraph.

7) Furthermore, at pag 11 (7th line from the bottom) two references: Berry-Kravis 1992 and Maurin 2018 are wrongly cited. The right references (considering the sentence written by the authors) should be Berry Kravis et al., Nature Medicine 2021 and Gurney et al, Scientific Report, 2017.

We thank the reviewer for pointing this out, however, both citations are in fact correct. We refer to the cyclic AMP pathway dysregulation in FXS which is described in both references.

8) Minor point:

Several typos are present, e.g. Pag 13 …individual genes. And some…

We thank the reviewer for pointing out that this sentence was not clear, and have rephrased it in the current version.

9) I do not understand the sentence: Pag. 12: The neuron specific downregulation of FMRP targets upon loss of FMRP is therefore potentially the result of higher levels of FMRP protein expression.

We thank the reviewer for pointing out this confusing sentence. What this sentence meant to express is redundant as the one two sentences before it, i.e. "*We hypothesize that this neuron specific downregulation of FMRP targets is due to the neuron specific expression of FMRP itself, and the loss of FMRP has a stronger impact on a subset of FMRP bound mRNAs*". Therefore here we removed the sentence in question to reduce redundancy.

Reviewer #2:

In general, I am satisfied with the revision.

We thank the reviewer for previous constructive comments.

Reviewer #3 (open questions only):

> *"We examined differential expression between Fmr1-KO and WT mice for each cell type identified. In total, we identified 1470 differentially expressed (DE) genes (FDR < 0.01 and fold change >=1.15) in one or more cell types. The majority of DE genes showed small fold changes (mean fold change = 1.3x)." This reviewer is most concerned about getting conclusion with the DE with fold change >=1.15. With such a small fold change, many of them just could be from biological sample or experimental batch variations.*
>
> *"The fascinating observation of ours and other studies is that loss of FMRP does not cause a large effect on the mRNA levels of most genes, however, the effect is consistent across most genes within affected pathways. We did not try to make claims about any particular gene and in fact, we agree with the reviewer in that conclusions about any particular gene may be challenging at this level, and only applied this low threshold to define the numbers in Figure 2a. Our analysis instead focused either on specific genes with larger fold changes or on pathways or gene sets with similar function where the overall trend is highly significant. The overall small effect size of FMRP loss was one of the motivations of carrying out a single cell analysis of FXS loss of function. In this version we have made an effort to better make this point in the introduction: that the small effect size of the transcriptional changes detected is expected, given that more robust changes would have been observed also by previous studies which relied on bulk analysis of gene expression."*

>> I understand that there could be masking effect in overall change by FMRP loss in bulk RNAseq. However, with shingle cell RNAseq, in specific cell types, it is more probable to have larger effect, that is why single cell analysis is needed.

We thank the reviewer for helping us clarify the significance of the small fold change, which is an important point:

1. The first concern addresses the biological significance of these changes. Very mild changes in the molecular landscape in the nerve system can lead to profound disease. For instance, in the 2019 paper published by the Tsai team (Mathys et al, 2019), the authors investigated the single cell RNA profile in human brain tissues from individuals that suffered from Alzheimers' disease. Notably, the Alzheimer's brains suffer from neuronal death, while the FXS neurons are otherwise healthy in comparison. It is conceivable that the Alzheimer's brain should show stronger transcriptomic changes. Nevertheless, the transcriptome changes detected in human Alzheimer's brain cells were also very mild, similar to our data, and the criteria used by Mathy's *et al.* for selecting differentially expressed genes reflects these low fold changes: "*only genes that are significantly supported by both models using the criteria FDR-corrected P < 0.01 in two sided Wilcoxon-rank-sum test, absolute Log-fold > 0.25, and FDR-corrected P < 0.05 in Poisson mixed model are considered*" (Log-fold > 0.25 = fold change > 1.19).
2. Another concern of the reviewer is on the technical level. The reviewer questions that, while we try to argue for the power of single cell RNA-seq, why we did not observe stronger changes. It is indeed thanks to the power of single cell RNA-seq, we could pick up such mild changes in a cell specific manner. When looking at the bulk RNA-seq using the combined expression for FMRP-KO or WT cells in our data, we could detect only a very small number ofgenes with significant changes, with the exception of Fmr1 due to the Knock-out (Fig S2).

In summary, it is a biological reality that in a lot of neurological diseases and their models, transcriptome changes are widespread and very mild. Facing this challenge, single cell RNA-seq enabled us to detect cellular changes that were otherwise undetectable using traditional bulk RNA-seq. Having said this, we agree with the reviewer on the hesitancy in trying to interpret such small changes for individual genes. And it is precisely for this reason, that we have focused on gene set enrichment analysis throughout the manuscript.

> *"We want to stress that our approach to look at functionally related genes using gene set enrichment analysis (GSEA) does not rely on a cutoff for selecting differentially expressed genes, but instead examines the fold changed ranked gene list to identify any coordinated expression change involving genes that belong to the same pathways or categories. As a result, the genes in the pathways identified show a consistent (and significant compared to other lists of genes) up or down regulation, which is a strong indicator of a biologically relevant change resulting from the loss of FMRP."*

>> I understand the author's claim, but I still do not buy it fully. With the sets with statistically insignificant genes even with high fold change rank, the further analysis of GSEA would not be meaningful. Throughout the entire manuscript, it should be clearly stated that the criteria for the selected gene sets used for GSEA.

We apologize if the rationale for performing this analysis was not clear in our previous response. We chose to carry out a GSEA rather than a differential gene expression analysis (DGEA) because the general small effect size indicated that the most relevant effects of FMRP loss, if they existed, would impact certain processes or pathways rather than any one gene in particular. GSEA is ideal for this task, and in fact when it was proposed, the authors showed that GSEA had power to detect expression changes that affected a process even with minimal power to detect the effect on the individual genes that were members of the pathway. Here is a quote from the seminal paper by Subramanian et al (Subramanian et al.

2005): "*The method revealed that genes involved in oxidative phosphorylation show reduced expression in diabetics, although the average decrease per gene is only 20%. The results from this study have been independently validated by other microarray studies (5) and by in vivo functional studies (6)*". This was similar to the situation we found ourselves in when we obtained the data described in Donnard et al. GSEA does not use filtering, it tests whether the statistics in question (fold change between FMRP-KO and WT) distributes differently for genes that belong to a predefined process compared to all genes assayed. In the paper we focused on the **processes** that showed significant change in either direction based on GSEA, not DGEA. In addition, we did not pre-select any of the pathways that would be tested on the analysis, and used the entire set of categories present in Gene Ontology, filtering out only those with fewer than 30 genes or more than 3000 genes.

> *3. Given the nature of the data with such a small DE FC, the authors should show separate tSNE plots for Wt versus KO in all tSNE related figures. It wonders whether there is no distinct cell cluster(s) in fmr1 KO compared to control. Even with overall similar presentation in tSNE plot, Wt and KO could show some differential proportion change better if they are presented separately.*
>
> *"The reviewer raises a valuable question, we did in fact provide a figure of the tSNE representation of the cells colored by their genotype in Figure S1b. As we discussed in the text, there were no observable differences or clusters that were formed by cells of only one genotype. This is not encouraging, considering the analysis relies on a set of genes that exhibit the highest variability across cells, and this is largely dominated by the cell type specific signal. Likewise, we examined these genotype plots for every subclustering analysis performed, using each cell type independently, and found no consistent differences that signalled a genotype difference in proportions. Again, this is not surprising given that the changes we observed in expression are moderate. We provided both the code to generate these plots (https://github.com/elisadonnard/FXSinDrop) as well as the single cell matrix used as input (GSE147191)."*

>> It was asked to present separate tSNE plots of wt versus fmr1 KO, not overlapped in different colors to show clear gene expression change in each cell types in single cell analysis.

We thank the reviewer for clarifying their suggestion, and have included the separate tSNE plots in Figure S1 and S14.

> *6. In Figure 4a, if we look at the terms with FDR<0.05, excitatory and interneuron do not show much difference.*
>
> *"We thank the reviewer for making an insightful observation. It is indeed our intention to show that for the synaptic related GO terms (Figure 4a), excitatory and inhibitory neurons behave very similarly. This is intended to contrast with the divergent behavior of the excitatory and inhibitory neurons for translation and mitochondria related GO terms (Figure 4b-c). We intend to show GO terms that behave similarly and differently to present this overview that excitatory and inhibitory neurons share some common responses but also have some divergent responses. However we have moved Figure 4a to 4c, and modified the text of this section to further clarify the intention."*

>> However, it seems that the excitatory and interneurons seem to have a bit specific trend. For example, significant excitatory GO terms are more in synapse and significant interneuron GO terms are more specifically in synapse assembly, not just synapse.

We thank the reviewer for clarifying this point. Indeed, there is a difference in which specific synapse-related GO terms show the strongest signal in these two neuron types, and we have clarified the text in the manuscript to avoid giving the impression that the changes are the same. The different changes in these subtypes are also detailed in the following paragraph that discusses the genes with strongest downregulation in each subtype.

> *8. ìThe cell type specific alteration of the transcriptome is a sensitive reflection of the cellular status, and can serve as a first step towards an overview of the molecular impact of FXS.î If this is the purpose of this manuscript, cell-type specific KD of Fmr1 including neuron, endothelial cell and astrocytes should be shown. It is likely that the observed DE patterns of this manuscript is combined effects of cell intrinsic and extrinsic functions of Fmr1 and/or combined effects of multiple cells lacking Fmr1. Some experimental verification that can support the authorsí conclusions should be presented. Otherwise, enthusiasm on this manuscript would be minimal.*
>
> *"We thank the reviewer for affirming the significance of our data as a sensitive reflection of the cellular status for the mouse model of FXS. The pathophysiology of the FXS is complicated and involves the interaction of numerous cell types and pathways. Our data presents the first of such with an overview, and we believe it will prove a valuable resource for the FXS field, the field of neurodevelopmental diseases, as well as the field of neurodevelopmental biology as a whole. And, as with the FXS disease, FMRP is lost in all cell types, the cellular status reflected by the transcriptome changes could be due a combination of both intrinsic and extrinsic disturbances. Indeed it would be an important next step to compare the transcriptomic changes in cell type specific KO models, in order to parse out the cellular intrinsic and extrinsic mechanisms. We agree that validation of our conclusions would increase the impact of our study, however, given that this is the first study that dissects cell type specific effects on mRNA levels in-vivo for the Fmr1-KO mouse, we believe that validation work will be critical and we are sure that this study will motivate them. Nevertheless, we thank the review for this insight and have added this point to the Discussion."*

>> This was not addressed properly. At least, the single, most significant finding of their claims could be validated.

The most significant finding of our manuscript was the identification of cell type specific alterations, as the reviewer pointed out in the previous comment. We saw two strategies for validating this finding: 1) validating changes of single genes, and 2) validating changes of pathways.

For validating changes of single genes, we had foreseen the challenge in achieving this due to the small magnitude of most of these changes. Therefore we attempted to validate the protein level changes of Mt1/Mt2, the strongest changed genes in all the DE genes, which are downregulated in astrocytes (log2FC = -0.7), and are relevant to astrocyte-neuron communication. We tested two antibodies against MT1/MT2 in western blots with cortical tissue lysates or with primary cultured astrocyte lysates, as well as immunostaining in cortical slices. Unfortunately, the available antibodies were too unspecific to lead to any conclusions.

We did confirm the downregulation in Mt1 expression using RT-qPCR in independent cortical samples, as detailed in our previous response to Reviewer #2.

For validating changes of pathways, the most straightforward approach is to perform independent cell cultures and RNA-seq, although this approach will take away the cellular context in the organ. Nevertheless, in a related work of ours (Shu et al, 2020), we performed mouse neuron culture and RNA-seq, and showed a similar trend of downregulation of overlapping pathways comparing FMRP-KO to WT, although very few genes show a significant change individually. After we initially submitted our manuscript, the work by Utami et al. (Utami et al., 2020) was published using human FXS derived neurons and FMRP-KO neurons that also showed multiple overlapping neuronal pathways downregulated. We believe these two studies in both mouse and human systems, in our hands and in an independent lab, and in a different cellular context (cell cultures) in combination serve as a strong independent validation for the data in our manuscript. We have cited these two studies and discussed their implications in our manuscript.

> *13. The comparison between mouse brain and human should be with human post mortem or at least forebrain organoids at comparable developmental stage.*
>
> *"We agree with the reviewer that transcriptomic data from human FXS in vivo neurons, or single cell data from post mortem cortex, would be ideal for comparison. However, to the best of our knowledge, these data do not exist. However, in the past month (after our manuscript had been reviewed), Kang et al. published the first ever human FXS organoid model (Kang et al. 2021). Kang et al. present evidence for enhanced neuronal excitability through immunocytochemistry, and in agreement with our results they report downregulated neurodevelopmental pathway genes, as well as upregulated protein translation and mitochondrial pathway genes by single cell RNA-seq, albeit without defining the cell types in which such dysregulation happens. The conclusions by Kang et al. strongly corroborate our findings. In return, our data complements that of Kang's with unparalleled cell type resolution (for example, as shown in Fig 4, the excitatory and inhibitory neurons exhibit a change in opposite direction for translation and mitochondrial pathways, which Kang et al. mention only as upregulated). We believe our data combined with studies such as these will provide invaluable resources for the FXS research community. We have added the above to Discussion."*

>> Citation should be added.

We have added the relevant citation to the Discussion.

REFERENCES

Gholizadeh, Shervin, Sebok Kumar Halder, and David R. Hampson. 2015. "Expression of Fragile X Mental Retardation Protein in Neurons and Glia of the Developing and Adult Mouse Brain." *Brain Research* 1596 (January): 22–30.

Subramanian, Aravind, Pablo Tamayo, Vamsi K. Mootha, Sayan Mukherjee, Benjamin L. Ebert, Michael A. Gillette, Amanda Paulovich, et al. 2005. "Gene Set Enrichment Analysis: A Knowledge-Based Approach for Interpreting Genome-Wide Expression Profiles." *Proceedings of the National Academy of Sciences of the United States of America* 102 (43): 15545–50.

Till, Sally M., Lasani S. Wijetunge, Viktoria G. Seidel, Emily Harlow, Ann K. Wright, Claudia

Bagni, Anis Contractor, Thomas H. Gillingwater, and Peter C. Kind. 2012. “Altered Maturation of the Primary Somatosensory Cortex in a Mouse Model of Fragile X Syndrome.” *Human Molecular Genetics* 21 (10): 2143–56.
