# Supplementary material for: Single cell transcriptomics reveals dysregulated cellular and molecular networks in a fragile X syndrome model
Source: PLoS Genet. 2022 Jun 8;18(6):e1010221. doi: 10.1371/journal.pgen.1010221 (PMC9212148; doi:10.1371/journal.pgen.1010221)
Supplement: Attachment — Submitted filename: PLOSGenetics_response_to_reviewers_round3.pdf [file pgen.1010221.s021.pdf]

We thank the reviewers for their previous suggestions and address any outstanding comments below.

Reviewer #3:

1 and 8. This reviewer appreciates the authors' efforts, and understands that the authors tried to show meaningful change in trend of specific subset of genes by the lack of FMRP. but respectfully disagrees with the authors' claims. As stated, the mild changes of multiple functionally related genes could contribute to the overall effect of FMRP loss in the pathogenesis of FXS. However, such arguments would get meaningful if the authors could provide experimental evidence to support the functional change/deficit caused by FMRP loss. Without such validations, enthusiasm on this manuscript is still moderate. The authors mentioned in their response and the reference (5) and (6), in the case where the average decrease per gene was only 20%, but could be validated by microarray and in vivo functional studies. Instead of working on single genes Mt1/Mt2 which were selected based on differential gene expression level, the authors could perform a knockdown of several functionally related genes to Mt1/Mt2, which is relevant to astrocyte-neuron communication based on GSEA, and then analyze if this could indeed mimic FMR1 KO phenotype in astrocyte-neuron communication in specific cell types. This way, the authors' claim with GSEA could be fortified. It is not sure this manuscript without functional validation as is satisfies the PLOS Genetics criteria for publication in terms of substantial evidence for its conclusions.

We wholeheartedly agree with the reviewer in that experimental confirmation of the functional impact of the changes we report would greatly enhance the impact of the paper. However, *in vivo* experimental validation such as the reviewer suggests would be quite involved taking up to a year of work. In our view, validation involving microarrays to confirm the decrease observed in gene expression is unnecessary given robustness of single cell RNA-Seq and the reproducibility of the impact we observed in several functional pathways in other systems such as human cultured neurons. As such, we hope the editor agrees with us and the other two reviewers, that experimental validation is outside the scope of our study. As presented, our work presents an invaluable characterization of the early molecular signatures that underlie the FMRP-KO phenotype. As our colleagues have shown in human iPS derived neurons, FMRP loss results in a complex molecular phenotype that we are only beginning to appreciate. Our work highlights the key pathways that are affected in small yet consistent ways and that help explain how, together, they integrate into the debilitating cognitive effect that results.

We respectfully disagree with the reviewer, and believe that even without the additional experimental validation suggested, this manuscript satisfies PLOS Genetics criteria for substantial evidence. Our conclusions on gene expression effects in the absence of FMRP in neurons are substantiated by orthogonal studies based on human ESCs and iPSCs, and independent studies by us and others in the same mouse model at the bulk expression level which we refer to in the manuscript. More importantly, our study provides novel data and insights that have significant implications to the current prominent hypotheses and avenues of therapeutic intervention being pursued by the Fragile X and autism spectrum disorder research

community. Our analysis reveals a heterogeneity in the response of different cell types to the absence of FMRP that was never shown previously, including a broad effect of downregulation of known FMRP bound mRNAs that is specific to neurons. We also detect changes in expression levels of cell signaling and surface protein encoding genes that point to a critical role of astrocytes in generating an environment of increased neuronal excitability and that can be further explored to determine the extent of their involvement. We believe this resource will have a strong impact on our understanding of Fragile X and will be of broad interest to PLOS Genetics readers.

13. As reviewer #1 pointed out that some of papers mentioned in the text or in the supplementary material do not appear in the final list, the authors should take a look at the missing references carefully. For example, newly added Kang et al. is missing in the reference list.

We thank the reviewer for pointing out this missing citation and have included it in the updated manuscript, as well as revised the remaining references to ensure no other issues could be found.
